# Accuracy of computed tomography perfusion in assessing metastatic involvement of enlarged axillary lymph nodes in patients with breast cancer

**DOI:** 10.1186/bcr1738

**Published:** 2007-07-05

**Authors:** Yun Liu, Massimo Bellomi, Giovanna Gatti, Xuejun Ping

**Affiliations:** 1 Ningxia Medical College Hospital, Yinchuan, Ningxia, 75004, China; 2 Department of Radiology, European Institute of Oncology and School of Medicine, University of Milan, Italy; 3 Department of Senology, European Institute of Oncology, Milan, Italy

## Abstract

**Introduction:**

The purpose of this study was to evaluate the diagnostic accuracy of computed tomography (CT) perfusion in differentiating metastatic from inflammatory enlarged axillary lymph nodes in patients with breast cancer.

**Methods:**

Twenty-five patients with 26 locally advanced breast tumors and clinically palpable axillary lymph nodes underwent dynamic multi-detector CT (LightSpeed 16; General Electric Company) at one scan per second for 150 seconds at the same table position after 40 ml intravenous contrast injection at 4.0 ml/second. Semi-automatic calculation of values of perfusion parameters – blood flow (BF), blood volume (BV), mean transit time (MTT), and permeability surface (PS) – was performed. Results were compared with pathology and with Her-2/neu and Ki-67 levels in a surgical specimen of the primary tumor.

**Results:**

Examined lymph nodes were inflammatory in 8 cases and metastatic in 18. Mean values of perfusion parameters in inflammatory and metastatic nodes, respectively, were BF of 76.18 (confidence interval [CI], 31.53) and 161.60 (CI, 40.94) ml/100 mg per minute (*p *< 0.05), BV of 5.81 (CI, 2.50) and 9.15 (CI, 3.02) ml/100 mg (not significant [n.s.]), MTT of 6.80 (CI, 1.55) and 5.50 (CI, 1.84) seconds (*p *= 0.07), and PS of 25.82 (CI, 4.62) and 25.96 (CI, 7.47) ml/100 mg per minute (n.s.). Size of nodes, stage of breast cancer, Ki-67 and Her-2/neu levels in breast cancer, and expression of primary tumor activity were not correlated to any perfusion parameter in metastatic nodes.

**Conclusion:**

CT perfusion might be an effective tool for studying enlarged axillary lymph nodes in patients with breast cancer. It gives information on vascularization of lymph nodes, helping to understand the changes occurring when neoplastic cells implant in lymph nodes.

## Introduction

Metastatic spread of tumor cells is responsible for the majority of cancer deaths. Tumor cell dissemination is mediated by a number of mechanisms, including local tissue invasion, lymphatic spread, hematogenous spread, or direct seeding of body cavities or surfaces. Clinical and pathological observations have strongly suggested that, for many tumors, the most common pathway of initial dissemination is via the lymphatics, with patterns of spread via afferent vessels following routes of natural drainage [1-3]. Cancer characterization, choice of treatment, and prognosis are strictly linked to the lymphatic local spread, lymph node metastases, and distance metastases. At present, there is no accepted accurate imaging modality or technique for the diagnosis of lymph node metastases [4-6] and this is one of the major limits of imaging techniques in tumor staging.

Functional imaging techniques, including dynamic contrast-enhanced computed tomography (CT), are an emerging tool proposed to evaluate blood supply and kinetics, to act as an *in vivo *marker of neo-angiogenesis, and to help diagnose tumors and monitor tumor response [7-9]. Functional data provided by CT perfusion (CTp) parameters and the related functional maps may add some diagnostic value to the simple morphologic CT images and increase the accuracy in diagnosing metastatic lymph nodes.

The aims of this prospective study were to evaluate the diagnostic accuracy of CTp in the differential diagnosis of benign or malignant enlarged axillary lymph nodes in patients with breast cancer and to correlate CTp parameters with the index of tumor aggressiveness, presuming that CTp mirrors angiogenetic activity.

## Materials and methods

### Subjects

This study was approved by our Institute Ethics Committee, and written informed consent was obtained from all patients. The inclusion criteria were patients with cytology- or histology-proven breast cancer, candidates to surgery with axillary dissection, and the presence of at least one palpable axillary lymph node that was well defined and easily clinically identifiable. This node was defined as the 'target' node in our research. Twenty-five consecutive patients who met the inclusion criteria were prospectively enrolled (one patient had bilateral breast cancer and bilaterally enlarged axillary lymph nodes). The cutaneous projection of the target node was marked on the skin with an indelible felt pen. The mean age was 55.4 years (range, 36 to 79 years). All patients were women.

### Computed tomography perfusion technique

CTp was performed as an additional examination for patients within 48 hours prior to surgical axillary nodal dissection. CTp was performed using a 16-slice multi-detector CT scanner (LightSpeed 16; General Electric Company, Fairfield, CT, USA). Table position at the level of the marked target node was recorded while defining the scan range by the external laser alignment light. A preliminary non-contrast 2.5-mm-thick CT of the axillar region (from the supraclavicular fossa to the diaphragmatic muscle cacumen) was performed to guarantee a panoramic view of the entire anatomical region and thus to help the recognition of anatomical landmarks. The target node was identified on the images, verifying the alignment of the table position with that recorded when centering the patient. A scanning range of 20 mm was selected for the dynamic CT to include the maximum volume of the target node. Dynamic study of this volume was performed (120 kV and 200 mA) reconstructing four contiguous 5-mm slices and scanning at the same table position at 1 second gantry rotation time, with 150 seconds total duration and no delay, after injection of 40 ml of non-ionic iodinated contrast medium (Iomeron 400 mg of iodine/milliliter; Bracco Imaging SpA, Milan, Italy) at 4 ml/second, followed by 40 ml of saline at 2 ml/second, via an 18- to 20-gauge cannula in the contralateral antecubital vein (in the right arm of the patient with bilateral breast cancer). After contrast material administration, six dynamic CT acquisitions were performed with breath-hold at the following intervals: 0–35, 40–45, 50–55, 60–65, 70–75, and 120–150 s. The timing and intervals were chosen to obtain a high number of measurements in the increasing part of the enhancement curves, which has a high and fast variability, whereas a smaller number of measurements is required in the washout phase, where these changes are slower. During each of the six dynamic perfusion series, the women received a local dose of 20 mGy and an effective dose of 1 mSv. With Montecarlo simulation software (CT-Expo, Medizinische Hochschule/Experimentelle Radiologie, Hannover, Germany) [10], the estimated dose to the breast was set at 0.3 mSv per series and 1.8 mSv for the entire study. A standard reconstruction algorithm with no edge enhancement was used for dynamic scanning.

### Image data analysis

**Figure 1:**
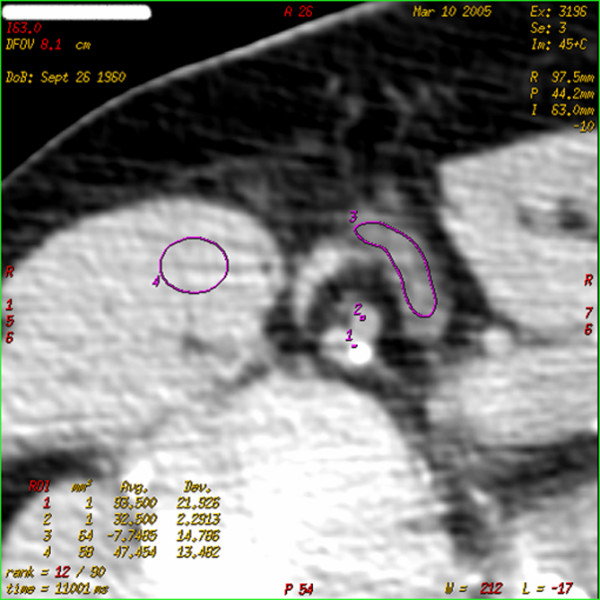
Image from one of the slices obtained by dynamic computed tomography centered on the palpable axillary node in patient AF with right breast carcinoma. A region of interest (ROI) was manually drawn along the margins of the target node (3). ROIs 1 and 2 were positioned in the axillary artery and vein. They were chosen with a very small area, as suggested by the software designer, to avoid partial volume effects. ROI 4 was drawn in the Teres major muscle for control.

Images and data obtained were transferred to an image-processing workstation (Advantage Windows 4.2; General Electric Company) and analyzed by a single expert reader (2 years of experience on CTp), who was blinded to pathology results. Commercially available software (CT perfusion 3; General Electric Company) was used for perfusion CTp analysis. In one of the four slices, a region of interest (ROI) small enough to avoid partial volume effects (2 to 6 pixels) was placed in the aorta (or subclavian artery or axillary artery when the aorta was not included in the section) for calculation of the arterial input, and a circular ROI (area range, 100 to 300 mm^2^) was placed in the Teres major or subcapsularis muscle for internal standard reference control. A third ROI was hand-drawn along the visible margins of the node (Figure 1). The software provided automated calculation of the following perfusion parameters in the ROI areas: blood flow (BF) in milliliters per 100 mg per minute, blood volume (BV) in milliliters per 100 mg, mean transit time (MTT) of the contrast medium through the ROI area in seconds, and permeability surface (PS) area product, which measures contrast medium extravasation into the extravascular space, in milliliters per 100 mg per minute. Values of perfusion parameters were averaged across three or four slices; when the target node was not visible in one slice, the set of slices was eliminated.

### Pathological evaluation

The target node was identified by the surgeon before the skin incision and marked by a suture on the specimen. Tumor tissue and node specimens were fixed with 10% buffered formalin and embedded in paraffin. The lymph nodes were bisected along the major axis and were sliced at 3- to 4-mm intervals before being fixed in 10% neutral buffered formalin for 6 to 8 hours under vacuum at 37°C. All the available tissue slices were then dehydrated, cleared, and embedded in paraffin. From the paraffin blocks, pairs of sections were cut at 50-μm intervals until complete sectioning of the nodes was achieved. One section of each pair was routinely stained with hematoxylin and eosin, whereas the mirror sections were kept unstained. The metastatic foci up to 2 mm in greatest dimension were reported as micrometastases; those larger than 2 mm define the node as metastatic.

Her-2/neu and Ki-67 levels were performed in the surgical specimen of the primary tumor; a polyclonal antibody for Ki-67 (Dako Denmark A/S, Glostrup, Denmark) was used at 1:350 dilution. Sections for Ki-67 were microwave-treated in 0.01 M citrate buffer (pH 6.0) at 750 W for 10 minutes to enhance antigen retrieval.

### Statistical analysis

A descriptive analysis of BF, BV, MTT, and PS was performed; mean values, standard deviations, and confidence intervals of metastatic and inflammatory nodes were calculated. The differences between BF, BV, MTT, and PS values of metastatic and inflammatory lymph nodes were evaluated applying a non-parametric Mann-Whitney *U *test for two independent groups. The linear correlation coefficient (Pearson's *r*) was calculated to estimate the correlation between CTp values and Ki-67 or Her-2/neu levels in the primary breast tumor.

## Results

Eleven out of 25 patients had right breast cancer, 13 had left breast cancer, and 1 had synchronous bilateral breast cancer. The locations of the 26 primary breast tumors were upper outer quadrant (5), upper inner quadrant (7), lower inner quadrant (7), lower outer quadrant (4), and central (3). The primary breast carcinoma was invasive ductal carcinoma in 20 cases, invasive lobular carcinoma in 4 cases, and mixed invasive ductal and lobular carcinoma in 2 cases.

The 26 lymph nodes examined by CTp were all identified in the surgical specimen and at pathology. They were metastatic in 18 cases and negative for metastases in 8 cases, which were all diagnosed as inflammatory hyperplasia. The mean maximum diameter of examined nodes was 1.42 cm. The mean diameters of metastatic and normal nodes were 1.60 and 1.24 cm, respectively.

All acquisitions were successful and could be analyzed by the software. Values of perfusion parameters (± standard deviation) obtained from the ROI in the muscle were BF of 13.48 ± 5.35 ml/100 mg per minute, BV of 1.16 ± 0.65 ml/100 mg, MTT of 9.85 ± 3.49 seconds, and PS of 4.44 ± 3.26 ml/100 mg per minute.

At CTp, the mean areas of ROI drawn along the examined nodes were 12.9 cm^2 ^for inflammatory nodes and 23.2 cm^2 ^for metastatic nodes. The difference between the areas of metastatic and inflammatory nodes was not significant (*p *= 0.376).

**Figure 2:**
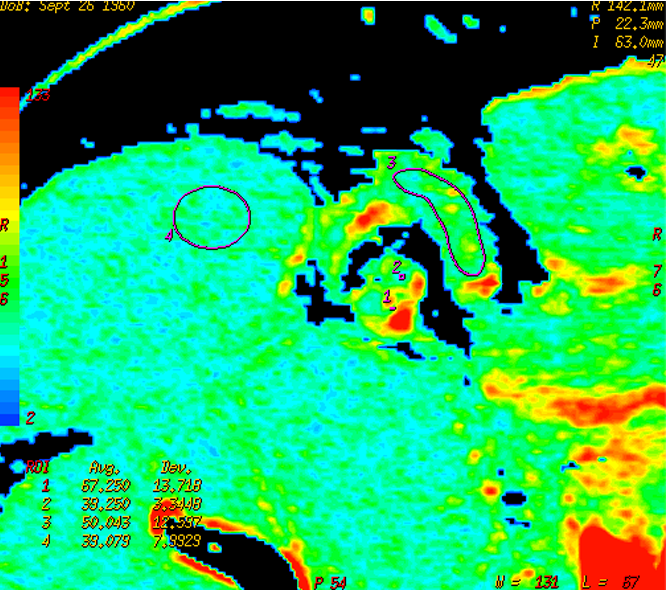
Map of perfusion of patient in Figure 1. The colored scale perfusion map designed using software shows how the perfusion of the target node is similar to that of the muscle. At post-surgical pathology, no metastases were found in the node.

**Figure 3:**
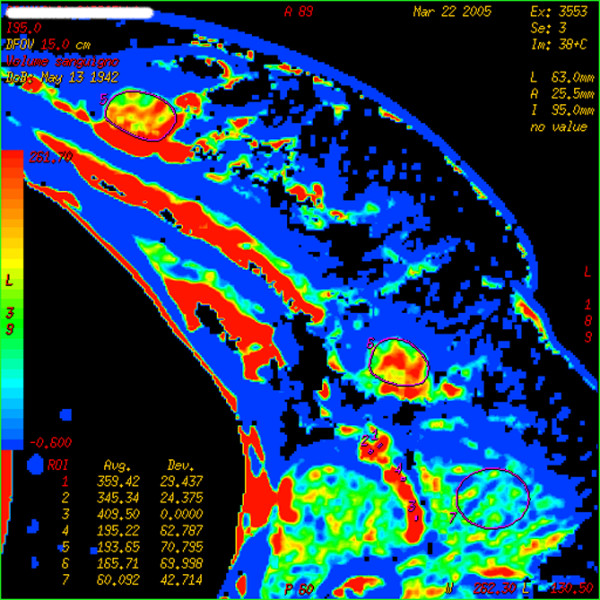
Colored map of blood volume (BV) in a patient with right breast cancer and omolateral palpable node. Both the primary tumor (outlined by region of interest [ROI] 5) and the target node (outlined by ROI 6) are visible on this slice. High values of BV are depicted both in the primary tumor and in the target node. At post-surgical pathology, the lymph node was determined to be metastatic.

BF, BV, MTT, and PS mean values of inflammatory (Figure 2) and metastatic (Figure 3) axillary lymph nodes are detailed in Table 1. The difference between the two groups is significant for measurements of BF, but not significant for those of other parameters. Of the 18 patients with metastatic nodes, 6 were staged as N_1_, 7 as N_2_, and 5 as N_3_; no correlation was found between any of the perfusion parameters evaluated in the target node and the N staging. In the same group of patients, the primary tumor was pathologically staged as T_1 _in 7 patients, T_2 _in 4, T_3 _in 6, and T_4 _in 1; the mean values of BF, BV, MTT, and PS in the four groups of patients showed no differences.

**Table 1:** BF, BV, MTT and PS mean values in inflammatory and metastatic axillary nodes

Parameter	Blood flow (milliliters per 100 mg per minute)		Blood volume (milliliters per 100 mg)		Mean transit time (seconds)		Permeability surface (milliliters per 100 mg per minute)	
	
Node	Inflammatory	Metastatic	Inflammatory	Metastatic	Inflammatory	Metastatic	Inflammatory	Metastatic
Mean value	76.18	161.60	5.81	9.15	6.80	5.50	25.82	25.96
Standard deviation	45.51	88.63	3.61	6.54	2.24	3.98	6.67	16.17
Confidence interval	31.53	40.94	2.50	3.02	1.55	1.84	4.62	7.47
*P *value	<0.05		0.1		0.07		0.84	

Mean values, standard deviations (SD) and confidence intervals (CI) of blood flow (BF), blood volume (BV), mean transit time (MTT) and permeability surface (PS) values for metastatic and inflammatory nodes. The *p *values of the Mann-Whitney *U *test are reported in the table. The Mann-Whitney *U *test proved a statistically significant difference between blood flow values of metastatic and inflammatory nodes (*p *< 0.05). However, the tests did not prove that the differences between the other parameters are statistically significant.

In patients with metastatic nodes, neither Ki-67 nor Her-2/neu levels in the primary breast cancer were significantly correlated to CTp parameter values (BF, BV, MTT, and PS).

## Discussion

Axillary nodal status is the most significant prognostic factor for predicting survival in patients with breast cancer even though many other factors such as tumor size, histological grade, S-phase fraction, DNA index, tumor ploidy, estrogen receptor and progesterone receptor status, and Her-2/neu and Ki-67 status have provided additional information to guide adjuvant therapy [11]. The accuracy of different imaging techniques in assessing axillary node status was extensively investigated. Ultrasound, implemented by power Doppler, showed a patient-based sensitivity of 86% and specificity of 93% [12]. Magnetic resonance imaging (MRI) with ultrasmall superparamagnetic iron oxide (USPIO) demonstrated a node-by-node sensitivity ranging from 100% to 73% and specificity ranging from 98% to 80% in the assessment of axillary node metastases and in pre-surgical staging of patients with breast cancer [13-15]. Nevertheless, clinicians consider the accuracy of imaging in defining lymph node metastases insufficient to drive the therapeutic decision and they prefer to base the type of surgery on the surgical biopsy and histology examination of axillary nodes in patients with breast cancer. Diagnosis of nodal status is generally obtained by sentinel node biopsy [16,17], but the evidence of palpable nodes contraindicates sentinel node biopsy and indicates total axillary dissection [18]. However, because of inflammation, a significant percentage of enlarged nodes (30% in our series, which is similar to figures reported in the literature [19,20]) are hyperplastic, and a correct pre-surgical diagnosis could spare unnecessary surgery that is associated with significant morbidity such as paresthesia, hematoma, seroma, restricted shoulder motion, and lymphedema [19].

The diagnosis of metastatic lymph nodes, which has an important clinical impact, is one of the main research fields in developing new imaging technologies or new contrast media. A recent study, based on ultrasound imaging of the primary tumor vascularization, showed that axillary involvement and cell proliferation are more frequent in highly vascularized tumors and that there seems to be a correlation between tumor vascularization and axillary node involvement [21]. Dynamic CT and perfusion parameters mirror tissue vascularization and might reflect angiogenic activity [22-24] and thus, theoretically, may be able to differentiate neoplastic from non-neoplastic tissue. A recent paper by Goh and colleagues [25] demonstrated that the inflammation of the intestinal wall is characterized by higher perfusion parameters than normal tissue, but by lower perfusion parameters than neoplastic tissue. The increased perfusion and permeability of inflamed tissue may be due to the effect of cytokines, which provokes vasodilatation and increases endothelial permeability, even if not at such high levels as neoplastic tissue, where angiogenesis plays an important role. The changes occurring in lymph nodes are probably different from those of solid tissues. The results obtained in our study show a significant difference of BF in metastatic and inflammatory enlarged axillary nodes. No significant differences were found in the other perfusion parameters (BV and PS), whereas the MTT values in metastatic nodes were lower than in inflammatory nodes (*p *= 0.07). We employed a commercially available software that was based on CT attenuation numbers obtained by a simple dynamic CT scan with intravenous (i.v.) injection of conventional contrast medium. The basic theory of CT perfusion software is based on the measure of blood supply to the ROI and its leakage into intercellular spaces. The changes induced by metastatic cells implanted in a lymph node are mainly expressed by blood vessel overgrowth and architectural disorganization [26]. Neoplastic and inflammatory infiltration have a similar behavior when invading extravascular space, and the difference between inflammatory and malignant changes is difficult to appreciate on the basis of criteria such as size [27] or even function of lymph node, as demonstrated by the similar pattern often observed by USPIO-MRI images [13,15,28]. This could be one of the reasons why we observed no differences in PS and MTT of metastatic and inflammatory enlarged axillary nodes: PS reflects the rate of leakage from the intravascular to extravascular-extracellular space within the capillary bed and MTT reflects the transit time through the vascular bed, being predominantly affected by the presence of shunts. Whereas in metastatic nodes BF is increased by newly developed vessels from angiogenetic activity, the changes in vascular endothelium and in the function of vessels induced by neo-angiogenesis and by cytokine-mediated inflammation are similar and values of PS and MTT are not different. Also, BV increases in metastatic nodes, but the difference does not reach statistical significance. Our results are in keeping with the observations by Yuen and colleagues [29], who described a higher CT contrast enhancement of metastatic axillary nodes than of non-metastatic ones: the simple measurement of density values after i.v. contrast medium administration were significantly different only after 1 minute, when metastatic nodes reached a density of 78.1 ± 27.8 Hounsfield units (HU) compared to 6.3 ± 32.7 HU of normal nodes. The early enhancement shown in their series of metastatic nodes probably reflects the higher blood supply due to angiogenesis, even if the simple protocol used was unable to study the contrast and blood dynamics and to draw significant conclusions. A recent paper by Bisdas and colleagues [30] reports no significant difference in CTp parameters between normal and metastatic nodes in patients with oropharyngeal cancer. Our data show higher values of BF in both inflammatory and metastatic nodes than those reported by Bisdas and colleagues. The difference in size and location of nodes might partially explain this difference. As speculated by Lurie and colleagues [31], the enhancement of lymph nodes starts in the subcapsular sinus and progresses to the medullar sinus; both neoplastic and inflammatory infiltration mainly occur in subcapsular spaces and the enlargement of this area may be one of the factors responsible for an increased blood supply, mirrored by an increase of BF.

The lack of a relationship between tumor biological activity and CTp values in metastatic lymph nodes is probably due to the different features investigated. Although both mirror tumor aggressiveness, tumor markers are associated with cellular proliferation, whereas CTp is probably related to the angiogenetic process.

The present study has some limitations. The series is limited and unbalanced (69.2% of metastatic nodes and 30.8% of inflammatory nodes), thus making it unreliable to evaluate the sensitivity and specificity of the results. The technique requires the identification of the node to be examined and the analysis is restricted to a range of 20 mm, thus preventing the examination of the whole axillary lymphatic bed. No data on completely normal nodes are available since it was impossible to identify small normal nodes observed at CT scan at subsequent surgery and pathological examination to confirm their normality. The unknown cardiac output of patients might influence the calculation of CTp values. The radiation dose of CTp might cause some concern for the contralateral breast, included in the scan field of view; the effective dose can be reduced by lowering x-ray tube energy for the dynamic scan, as suggested by Miles and colleagues [32]. Finally, the physiopathology and dynamics of blood supply to normal, inflammatory, and metastatic lymph nodes are not yet thoroughly understood and further studies on this topic and its relationship with CTp are needed.

## Conclusion

Our study suggests that CTp may provide useful information on lymph node status, adding functional to morphologic data. The use of multi-detector CT allows diagnosticians to reach a high spatial definition, and information on vascularization provided by CTp could be integrated with that of other diagnostic techniques to better understand the changes occurring in lymph nodes following neoplastic cell implantation and development.

## Abbreviations

BF = blood flow; BV = blood volume; CT = computed tomography; CTp = computed tomography perfusion; HU = Hounsfield units; i.v. = intravenous; MRI = magnetic resonance imaging; MTT = mean transit time; PS = permeability surface; ROI = region of interest; USPIO = ultrasmall superparamagnetic iron oxide.

## Competing interests

The authors declare that they have no competing interests.

## Authors' contributions

YL contributed to the study concept and design, data acquisition, manuscript drafting and editing, approval for important intellectual concepts, and literature research. MB contributed to the study concept and design, statistical analysis, manuscript drafting and editing, approval for important intellectual concepts, and data analysis and interpretation. GG contributed to the clinical study, data acquisition, literature research, and manuscript drafting and editing. XP contributed to the clinical study, data acquisition, literature research, and manuscript drafting. All authors read and approved the final manuscript.
